# Terrestrial Bioinspired Robots: Biological Models, Locomotion Modes and a Review of Technological Progress

**DOI:** 10.3390/s26216991

**Published:** 2026-11-05

**Authors:** Liang Wu, Shengyong Xu

**Affiliations:** Key Laboratory for the Physics and Chemistry of Nanodevices, School of Electronics, Peking University, Beijing 100871, China; 2301112135@stu.pku.edu.cn

**Keywords:** terrestrial bioinspired robots, dimensionless kinematics, gliding robots, actuation and sensing, technology readiness, morphological computation

## Abstract

Terrestrial bioinspired robots emulate the locomotion strategies of land animals and have potential applications in disaster response, planetary exploration, and inspection of complex terrain. This review examines the biological basis of six representative locomotion modes: multi-legged walking and running, elastic jumping, ground-coupled gliding, interface-mediated climbing, peristaltic crawling, and adaptive morphogenesis. It introduces dimensionless descriptors of their kinematics, with explicit reference scales for periodic motion and one-off events. We use morphology-function correspondence to compare representative multi-legged, quadrupedal, jumping, gliding, climbing/peristaltic, and multimodal robots with respect to mechanisms, actuation, sensing, control, quantitative performance, and application scenarios. The cited demonstrations range from laboratory mechanisms to selected quadrupedal field applications; their heterogeneous validation records do not support class-wide numerical readiness ratings. Incomplete reporting of sensing and endurance also limits comparison. The review identifies task-specific co-design needs, with sensing architectures matched to the contact and transition variables that must be regulated in each locomotion mode.

## 1. Introduction

Most human activity and robotic intervention take place in terrestrial environments. Wheeled and tracked systems are efficient on prepared surfaces, but their traversability decreases sharply on rugged mountains, collapsed rubble, and deformable soil. Terrestrial organisms, from mammals to arthropods, have evolved ways to traverse such terrain over hundreds of millions of years. Their locomotion strategies provide design principles for addressing some limitations of conventional mobile platforms.

Recent reviews have examined terrestrial bioinspired locomotion from several complementary perspectives. Mo et al. [1] compared biological jumping mechanisms and robotic strategies for takeoff, aerial righting, landing, and resetting, including actuator and energy-storage choices and reported jumping performance. Chen et al. [2] organized soft crawling robots by their actuation mechanisms and compared the associated designs, locomotion strategies, and performance limitations. For legged systems, Carpentier and Wieber [3] reviewed contact planning and dynamically feasible motion generation, emphasizing trajectory optimization and model predictive control. Zhou et al. [4] addressed soft-robot actuation and sensing directly, covering proprioceptive and tactile sensing, surface and internal sensor integration, and sensor-based closed-loop control. More recently, Yuan et al. [5] classified multimodal soft locomotion according to active control, reconfiguration, and environment-responsive mechanisms. Together, these reviews provide substantial coverage of individual locomotion modes, control methods, and enabling technologies.

A cross-mode comparison still needs to connect these perspectives: reported speed, jump height, or attachment force is difficult to interpret without the associated reference scale, sensor arrangement, actuation constraints, and test environment. The present review relates six selected locomotion modes to their biological mechanisms, the physical states relevant to feedback, and representative rigid, soft, and hybrid implementations. Its contribution is the joint treatment of reported sensing and actuation arrangements, mode-specific dimensionless descriptors and the information needed to calculate them, and the validation context of each demonstration. This is a bounded narrative synthesis, not an exhaustive survey or a universal performance ranking. Normalization is used only where its reference quantities are available, and laboratory, relevant-environment, and field demonstrations are distinguished without assigning one numerical technology-readiness level to an entire locomotion class. Table 1 positions this approach relative to the emphases of the selected reviews.

We review terrestrial bioinspired locomotion while excluding bipedal and humanoid systems and sustained powered flight. The selected classes emphasize ground support, attachment, propulsion, or transitions between substrate-dependent modes. Ground-coupled gliding is included when an unpowered aerial segment forms part of a terrain-crossing task, with takeoff and landing coupled to terrestrial locomotion. Standalone aerial navigation is outside this scope. Submerged and microgravity examples are included only as mechanism or interface comparisons; their tests do not establish terrestrial deployment readiness. Bipedal and humanoid systems share several mechanical principles with the included classes, but their balance, manipulation, and application requirements warrant a separate comparison. This boundary does not imply that excluded systems are less bioinspired or less mature. Two research questions structure the review. RQ1 asks which biological principles are adopted across locomotion modes. RQ2 asks how effectively those principles have been translated into mechanisms, actuators, sensors, and control architectures, and how their performance has been verified. Section 2 summarizes the underlying mechanics and introduces configuration-independent descriptions and mode-specific dimensionless descriptors. Section 3 compares representative prototypes, their actuation and sensing architectures, control strategies, and engineering maturity. Section 4 discusses gaps in engineering readiness and directions for integrated co-design.

## 2. Biological Models and Locomotion Modes of Terrestrial Bioinspired Robots

Terrestrial bioinspired robots draw on locomotion strategies that organisms use to negotiate complex terrain. We group these strategies into six modes. For each mode, we first describe the biological mechanism and then identify variables for comparison across scales and configurations. Section 3 relates these categories to robotic implementations.

### 2.1. Walking and Running

Walking and running are among the most common forms of terrestrial locomotion in mammals, reptiles, and insects. Periodic limb oscillation drives body translation, while discrete foot-ground contacts provide support and propulsion. During walking, the center of mass rises and falls along an arc, which permits periodic exchange between gravitational potential energy and kinetic energy. This exchange underlies the high energetic efficiency of walking [6]. During running, the legs store elastic energy: each leg is compressed like a spring during stance and releases the stored energy at takeoff. The spring-loaded inverted pendulum (SLIP) template describes this behavior across species [7]. The lateral leg spring (LLS) template extends this description to the horizontal plane for insect turning and lateral stability. Lateral leg elasticity can stabilize lateral body motion without continuous neural feedback [8].

Quadrupedal gaits, including walking, trotting, and galloping, differ in their footfall sequences and are coordinated by spinal central pattern generators. Multi-legged insects can continue moving after losing one or more contacts because redundant supports provide intrinsic stability [8]. Full and Koditschek formalized these observations in the neuromechanical templates-and-anchors hypothesis. This hypothesis distinguishes low-dimensional global locomotion templates from high-dimensional musculoskeletal and neural anchor models. It clarifies the roles of neural and mechanical regulation at different speeds and provides a basis for hierarchical legged-robot control [9]. Hierarchical reinforcement learning can also identify task-dependent criteria for gait switching [10]. Biological muscle-bone tensegrity structures combine compliant impact absorption with adaptive stiffness and have inspired rigid-soft coupled limbs. Engineering examples include a quadruped with a Bricard-mechanism deformable torso and deployable compliant feet made from PEBA elastomer [11,12]. Bird-neck-inspired hyper-redundant mechanisms also inform the design of flexible, high-degree-of-freedom limbs [13].

### 2.2. Jumping

Jumping allows frogs, kangaroos, locusts, fleas, and Senegal bushbabies to clear large obstacles, evade predators, or capture prey. The main mechanism is the gradual storage and rapid release of elastic energy. Muscles contract over a relatively long interval to load tendons or other elastic tissues, which then release the stored energy at takeoff. The resulting instantaneous power can greatly exceed that of direct muscle contraction. Larger jumpers such as frogs and kangaroos also recover impact energy through elastic structures during landing, which improves the efficiency of repeated jumps. Frog-inspired mechanisms for traversing large obstacles have demonstrated efficient energy storage and release [14]. At smaller scales, organisms use bistable elastic structures. Multi-deformation origami pneumatic actuators can serve as compact power units for miniature jumping robots [15]. Rapid chemical-energy release in insects has also inspired combustion-driven microrobots. Their soft combustion actuators provide millimeter-scale robots with high peak power [16].

A complete jump comprises three stages: explosive force generation at takeoff, aerial attitude regulation, and impact attenuation at landing. Senegal bushbabies perform repeated branch-to-branch leaps. Because their jumps combine crouched energy storage with multidimensional aerial correction, they provide a useful model for highly dynamic monopedal robots.

### 2.3. Climbing

Climbing is locomotion on vertical or inverted surfaces. Common biological models include geckos, tree frogs, insects, spiders, and Virginia creeper. The primary mechanical requirement is to resist gravity. Biological attachment can be divided into four strategies suited to different operating conditions.

Dry adhesion in geckos relies on millions of micro- and nanoscale setae that generate van der Waals forces [17]. The mechanism requires no liquid medium, functions in a vacuum, and tolerates modest surface irregularity, but dust and sand markedly reduce its load capacity. Wet adhesion in tree frogs and crickets depends on capillary and viscous forces produced by secretions between the pad and the surface. It conforms better to rough substrates than dry adhesion but is sensitive to ambient humidity. Outdoor insects commonly use claw interlocking. Microscopic spines mechanically engage asperities in porous substrates such as rock and wood. This strategy compensates for the poor contamination tolerance of dry adhesives [18]. Hexapod climbers equipped with microspines have demonstrated stable locomotion on these surfaces [19]. Negative-pressure adhesion, exemplified by octopus suckers, generates large attachment forces through a pressure differential but requires a smooth, well-sealed contact interface.

A more recent bioinspired strategy imitates the growth-mediated adhesion of Virginia creeper. It progressively forms microsuction structures that adapt to fractured, uneven, and discontinuous walls and extend the range of climbable environments [20]. Variable-stiffness adhesive paws can maintain attachment on both rough and smooth surfaces in microgravity [21]. These attachment mechanisms differ in their strengths and operating limits.

### 2.4. Peristaltic Crawling

Peristaltic crawling is the main locomotion mode used by limbless or nearly limbless organisms, such as earthworms, inchworms, and snakes, in confined spaces. Coordinated axial deformation of body segments alternates contact with and release from the environment to produce net body displacement.

Earthworm locomotion follows a repeated contraction-anchor-extension-release cycle. Circular-muscle contraction lengthens and narrows a body segment, whereas longitudinal-muscle contraction shortens and thickens it; these actions are coordinated with anchoring [22]. Shi et al. showed that the nonsmooth, corrugated surface of an earthworm reduces muscular contraction loss and sliding friction during peristalsis [22]. Inchworms use a characteristic omega-shaped deformation. The midbody arches while the front end remains anchored and the rear end advances. This strategy works on both horizontal and vertical surfaces, and grooved guidance structures can improve the path accuracy of inchworm-inspired soft robots [23]. Snakes have a wider locomotor repertoire that includes lateral undulation, rectilinear crawling, and concertina motion. They therefore provide a primary model for continuum soft robots. Hydraulically driven rigid-soft integrated joints can substantially reduce energy consumption in underwater snake robots [24].

Peristaltic locomotion is well suited to narrow pipes and soil fissures, but is inherently slow. Research on bioinspired robots therefore focuses on optimizing actuation timing and developing anisotropic frictional surfaces.

### 2.5. Adaptive Morphogenesis and Composite Deformation

Adaptive morphogenesis is an engineering strategy inspired by organisms that change body shape or limb posture as environmental demands change. In robots, a limb can reconfigure from a load-bearing leg on land to a flattened propulsive fin in water. Changing limb cross-section and stiffness allows a single robotic body to alternate between support and propulsion. Multi-environment robots have demonstrated this strategy [25].

### 2.6. Gliding

Gliding differs from pure jumping because aerodynamic lift and drag shape the aerial trajectory. Here, gliding refers to an unpowered aerial segment within a ground-based locomotion task, rather than sustained powered flight or standalone aerial navigation. Flying squirrels are a representative gliding model. Their compliant patagium increases planform area, while movements of the limbs and tail regulate angle of attack, pitch, and direction. A stored-energy launch combined with passive or actuated aerodynamic surfaces can extend range and reduce landing impact relative to a purely ballistic jump [26,27].

The main states are horizontal and vertical position, speed, flight-path angle, angle of attack, and body attitude. Cross-platform comparisons should therefore report glide ratio, lift and drag coefficients, normalized launch speed, and landing impact energy. Jump height alone does not characterize gliding performance.

Walking/running, jumping, gliding, climbing, peristaltic crawling, and adaptive morphogenesis use different mechanical regulation strategies and impose different design constraints. The descriptors below make these differences explicit without requiring a common joint-level model. Section 3 then examines how the modes are implemented in multi-legged, quadrupedal, jumping, gliding, climbing/peristaltic, and multimodal robots.

### 2.7. Configuration-Independent Descriptions and Mode-Specific Dimensionless Variables

For the reporting conventions proposed here, the reference length L is fixed: the hip-to-ground distance in the stated nominal standing posture for gravity-dominated legged comparisons [28,29], and the undeformed longitudinal main-body length for gliding and body-deformation descriptors. The measurement endpoints and reference posture must be stated. L remains fixed during a morphology change or mode transition. Results based on different length conventions require renormalization before comparison. T denotes the interval between successive equivalent events of a periodic motion, such as consecutive contacts of the same limb or successive passages of the same strain-wave phase at the same material point. An actuator’s drive frequency need not equal the locomotor cycle frequency. The following cycle-based definitions apply only when such repetition is present:

$$T=\frac{1}{f}\;;\;\tau =\frac{t}{T}=ft$$

$${r_{c}}^{*}\left(\tau \right)=\frac{r_{c}\left(t\right)}{L}\;;\;\kappa^{*}\left(\tau \right)=L\kappa \left(t\right)$$

In these definitions, $r_{c}\left(t\right)$ denotes the center of mass or a fixed body reference point, and $\kappa \left(t\right)$ denotes a representative curvature. For one complete cycle, define the dimensionless net displacement and corresponding mean net-advance rate as follows:

$$\Delta^{*}=\frac{\left\|r_{c}\left(t+T\right)-r_{c}\left(t\right)\right\|}{L}$$

$$\bar{U}=\frac{\left\|r_{c}\left(t+T\right)-r_{c}\left(t\right)\right\|}{T}\;;\;U^{*}=\frac{\bar{U}}{Lf}=\Delta^{*}$$

For limb i in periodic motion, define the phase and contact duty factor as follows, with total contact duration measured within one cycle:

$$\varphi_{i}\left(t\right)=2\pi frac\left(\frac{t-t_{0,i}}{T}\right)\;;\;\beta_{i}=\frac{T_{contact,i}}{T}$$

In the phase definition, $t_{0,i}$ denotes the reference contact-event time for limb i, and $frac\left(x\right)=x-floor\left(x\right)$. These variables describe coordination independently of joint count and linkage dimensions. The commonly reported length-normalized rate $\frac{\bar{U}}{L}$ has units of $s^{-1}$ and is therefore not a dimensionless speed. For gravity-dominated legged dynamics, we use the gravitational time scale and Froude number below [28,29]. We also define the dimensionless cost of transport:

$$T_{g}=\sqrt{\frac{L}{g}}\;;\;Fr=\frac{{\bar{U}}^{2}}{gL}\;;\;CoT=\frac{E}{mgd}$$

Here, E is the energy consumed over a measured travel distance d>0, m is the total moving mass including payload, and g is gravitational acceleration. The dimensionless cost of transport (CoT) is therefore energy use per unit weight and distance; a lower value indicates less energy use under the stated test conditions. Comparisons must use the same energy boundary (for example, electrical input or mechanical work) and report the payload and travel-distance convention [29,30].

The Froude number compares the inertial force scale $\frac{m{\bar{U}}^{2}}{L}$ with body weight mg, making it useful for comparing gravity-dominated legged locomotion across sizes with a consistent characteristic-length convention [28,29]. Equal Fr alone does not establish complete dynamic similarity when relative geometry, compliance, friction, actuation, or terrain conditions differ.

A single glide, morphology change, or mode transition need not have a locomotor period or frequency. We describe its measured duration and progress separately from the periodic variables:

$$T_{\mathrm{evt}}=t_{1}-t_{0};\;\eta =\frac{t-t_{0}}{T_{\mathrm{evt}}};\;0\leq \eta \leq 1$$

$$T_{\mathrm{ref}}=T_{g}=\sqrt{\frac{L}{g}};T_{\mathrm{tr}}=T_{\mathrm{evt}}\;\mathrm{for}\;a\;\mathrm{transition}$$

Here, the event begins at t_0_ and ends at t_1_. For a glide, the interval starts at entry into the unpowered gliding phase and ends at landing; any preceding launch phase is reported separately. For morphogenesis, it starts with the transformation command and ends when the target morphology satisfies a stated tolerance. A multimodal transition additionally requires a declared criterion for stable operation in the new mode. Endpoint tolerances and settling intervals must be reported. Event duration is not converted into a gait frequency. For the gravity-referenced terrestrial comparisons proposed here, the reference time is the gravitational time scale, not a measured cycle or material response time. Dimensional durations must also be reported; this convention neither applies at zero gravity nor establishes dynamic similarity between different mechanisms.

For gliding, $T_{g}=\sqrt{\frac{L}{g}}$ provides a body-scale gravitational reference for a terrain-crossing task with terrestrial takeoff and landing, rather than an aerodynamic similarity scale. During the aerial phase, a convective time scale $T_{a}=\frac{l_{a}}{U_{\mathrm{air},\mathrm{ref}}}$ may be more appropriate, where $U_{\mathrm{air},\mathrm{ref}}>0$ is a stated air-relative reference speed and $l_{a}$ is the fixed main-body length L for trajectory comparisons or a specified wing chord c for wing-scale aerodynamic response. The chosen length, reference speed, and flight condition must be reported, and the aerodynamic scale is not evaluated when the required inputs are unavailable.

Table 2 presents a minimal kinematic representation and the main dimensionless variables for each mode. Here, configuration independence refers to the choice of observable states, such as contact timing, center-of-mass trajectory, body strain, aerodynamic state, or morphology state, rather than to a universal governing model. These states can be described without specifying joint count or actuator layout. The normalization remains mode-specific because gravity, attachment forces, strain propagation, and aerodynamics regulate different motions; a one-off transition also has no necessary cycle frequency. Comparisons therefore require compatible physical quantities, reference scales, and test conditions. The sensing entries identify states needed for feedback or evaluation and do not imply that every prototype measures them. We do not combine the descriptors into a cross-mode performance score.

Quantifying morphology–function correspondence across modes requires a task-specific mechanical definition, not geometry alone. Translational stiffness $\left(N\cdot m^{-1}\right)$, rotational stiffness $\left(N\cdot m\cdot {\mathrm{rad}}^{-1}\right)$, and flexural rigidity $\left(N\cdot m^{2}\right)$ describe different responses; each requires a defined deformation measure, loading direction, boundary conditions, and operating state, with passive mechanics distinguished from closed-loop effects. Nonlinear responses also require a stated operating point or range and a tangent or secant convention. Ratios such as $R_{K}$ compare like definitions under matched measurement protocols, while comparisons across robots require compatible reference scales and stated environmental, payload, speed, and actuation conditions. The reviewed studies therefore do not establish a universal numerical mapping from morphology or stiffness to locomotor performance across modes.

## 3. Representative Robot Classes, Technical Implementations, and Applications

### 3.1. Multi-Legged (Hexapod and Octopod) Robots: Static Stability and Load-Bearing Potential

**Figure 1:**
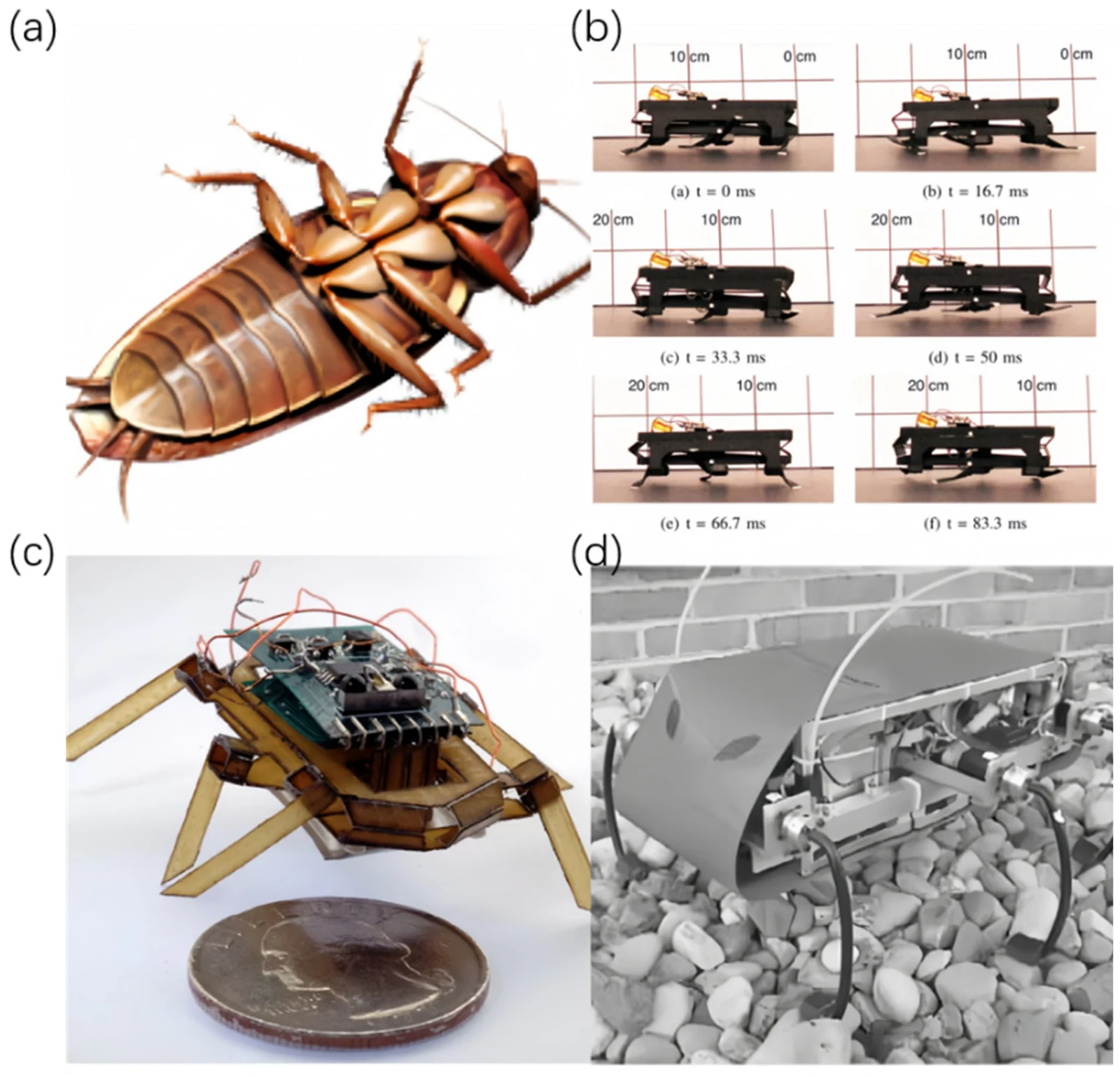
Cockroach-inspired multi-legged robots. (**a**) Biological model: the American cockroach; (**b**) time sequence of high-speed running by the DASH hexapod [31]; (**c**) the 2.4 g autonomous RoACH hexapod shown beside a coin for scale [32]; and (**d**) the RHex platform [34].

Multi-legged robots use redundant contacts, much like insects and crustaceans. The American cockroach in Figure 1a illustrates the biological model for the hexapods discussed below. If several feet lose contact or slip, the remaining feet can still stabilize the body, which is useful on boulder fields and earthquake debris. Research spans miniature laboratory prototypes, passive wheg-based platforms, heavy-duty terrestrial prototypes, and specialized deep-sea robots, each developed for different applications and at a different level of maturity.

Fearing’s group at the University of California, Berkeley developed the miniature DASH and RoACH hexapods using smart composite microstructures. DASH uses a single motor, a simplified kinematic design, and an alternating tripod gait to reach 15 body lengths per second; its compliant structure allows it to survive impacts after falls from a height [31]. Figure 1b shows the running sequence reported for DASH. RoACH achieves autonomous mobility with a very small mass budget [32], and Figure 1c shows the 2.4 g robot beside a coin for scale. Limited battery capacity, actuation power, payload, and endurance, however, keep both platforms focused on locomotion-mechanism studies rather than field engineering tasks [31,32]. Comparative studies have assessed hexapod designs in terms of structure, navigation, and control [33].

RHex, shown on rocky terrain in Figure 1d, uses six compliant rotary legs, each driven by one hip motor [34]. A clock-driven alternating-tripod gait coordinates the legs, while passive compliance accommodates ground contact. Its demonstrated intrinsic mobility does not require terrain sensing [34]. This architecture differs from the three-spoked Whegs mechanism discussed in Section 3.6. The GiAnt hexapod, developed for earthquake-debris scenarios, likewise traverses cluttered rubble [35].

**Figure 2:**
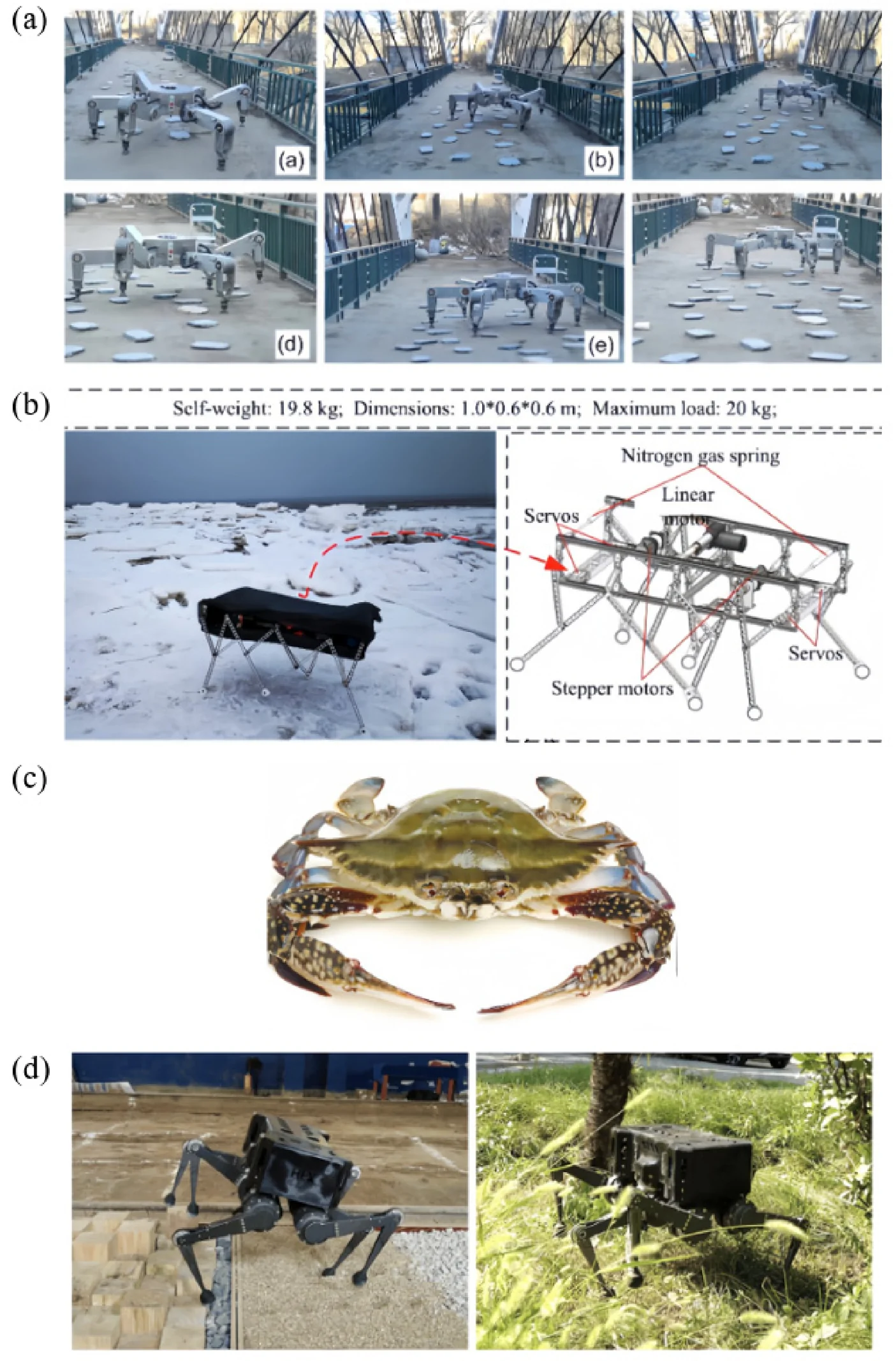
Multi-legged robots and their biological models. (**a**) ELSpider during heavy-duty field trials on rugged terrain [36]; (**b**) the Yanshan University HexaBeast with load-bearing Chebyshev-linkage legs [37]; (**c**) the crab biological model of RENS H3; and (**d**) the crab-inspired, lateral-walking RENS H3 hexapod [39].

Few heavy-duty terrestrial hexapods are available for mines or earthquake debris. Figure 2a shows the Harbin Institute of Technology platform on rugged terrain [36], whereas Figure 2b shows the Yanshan University platform with load-bearing Chebyshev-linkage legs [37]. Motion-planning and contact-force allocation methods improve payload capacity and slope stability on unstructured terrain, but both systems are research prototypes evaluated under specified terrain conditions [36,37]. For underwater applications, Shim et al. developed the arm-and-leg system of CRABSTER200, a six-legged seabed-walking robot. Its front limbs serve as both legs and manipulators, allowing the robot to combine seabed locomotion with object handling [38].

In 2025, Liu et al. introduced RENS H3 (Figure 2d), a hexapod inspired by crab locomotion (Figure 2c) and designed for lateral walking. Its hierarchical controller combines model predictive control with Cartesian foot-force compensation to adjust body pitch and roll in real time on unknown rugged terrain. In the reported tests, lateral walking provided better stability and lower energy consumption than conventional forward walking [39].

Technology readiness level (TRL) is interpreted using NASA’s criteria for the demonstrated system or subsystem, its configuration, and its intended operating environment [40]. TRL 3 denotes analytical and experimental proof of a critical function; TRL 4 denotes component or breadboard validation in the laboratory. TRLs 5 and 6 require component validation and system or subsystem prototype demonstration, respectively, in a relevant environment. TRL 7 requires a system prototype to function in its actual operational environment, whereas TRL 8 requires the completed system in its final configuration to be qualified through test and analysis. These distinctions concern evidence for a particular application. A quadruped’s field demonstration does not establish the readiness of a new gait or a different mission; a crawler’s demonstrated deformation and anchoring do not by themselves establish field readiness. The cited studies do not provide a uniform qualification record for each complete robot. We therefore compare reported validation stages rather than assign a single numerical TRL range to each locomotion class.

Heavy-duty terrestrial use remains underdeveloped despite progress in related applications. Mole-inspired forelimb diggers have been proposed for planetary soil sampling [41], and lightweight legged systems for lunar and Martian pits have reached proof-of-concept testing under low-gravity conditions [42]. Teams of multi-legged robots may also support wide-area scientific surveys on planetary analog terrain [43]. The cited demonstrations do not establish the readiness of dedicated heavy-duty equipment for sustained operation on rubble or boulder fields. The reviewed multi-legged systems span laboratory studies and terrain-specific tests; these heterogeneous demonstrations do not support a single class-wide TRL assignment.

### 3.2. Quadrupedal Robots: Dynamic Locomotion

**Figure 3:**
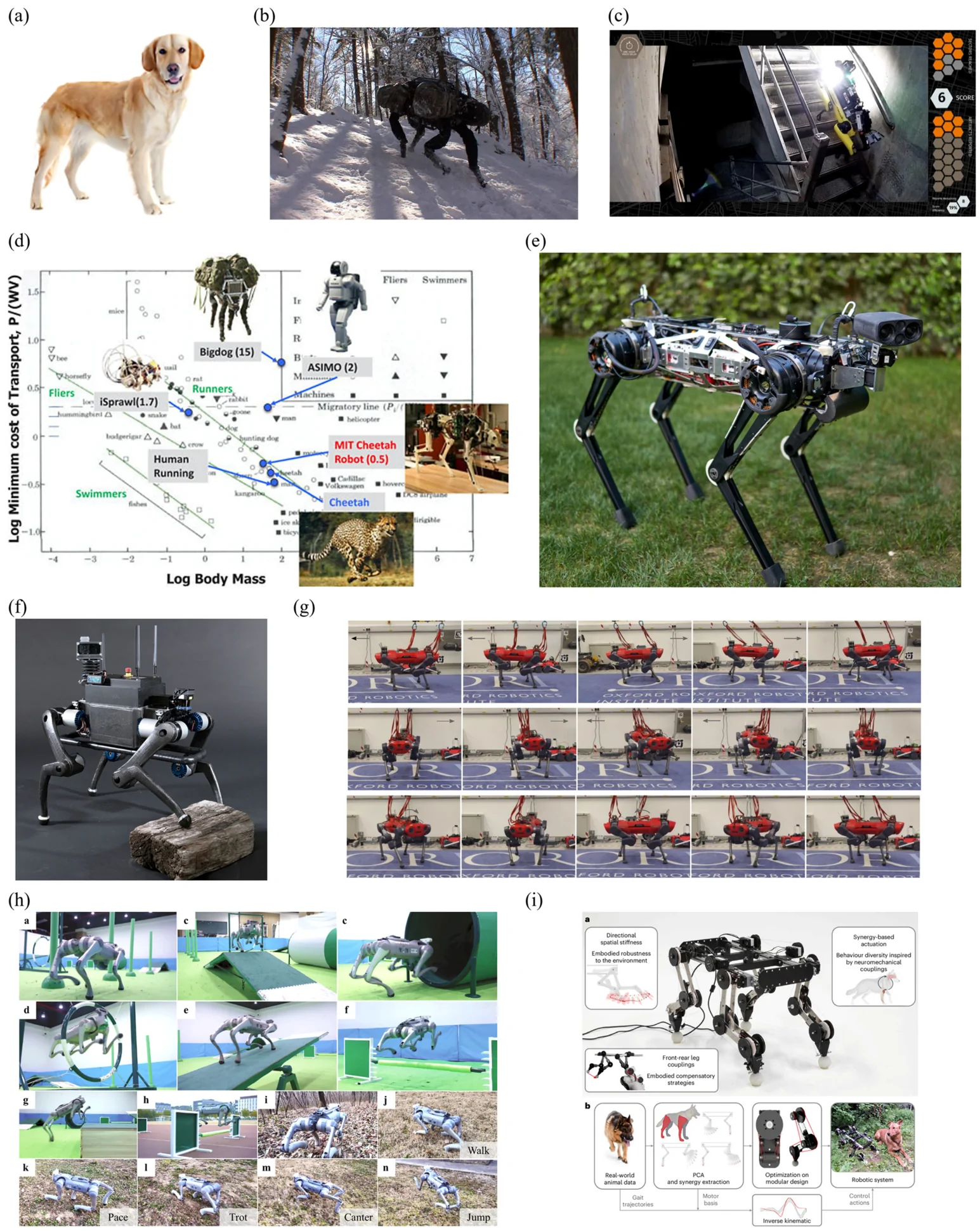
Quadrupedal biological models and robotic platforms. (**a**) Domestic dog; (**b**) BigDog during a hydraulic, heavy-load field test [44]; (**c**) Spot equipped for autonomous exploration [45]; (**d**) logarithmic comparison of cost of transport for terrestrial organisms and mobile robots [30]; (**e**) MIT Cheetah 3, inspired by the cheetah for energy-efficient high-speed running [47]; (**f**) the first-generation ANYmal platform [52]; (**g**) ANYmal C [53]; (**h**) Unitree Go2 on a randomized obstacle course [54]; and (**i**) the EPFL PAWS synergistically actuated quadruped [56].

Quadrupedal robots draw on mammalian body plans, exemplified by the domestic dog in Figure 3a. Among terrestrial bioinspired platforms, quadrupedal robots offer a favorable balance of speed, dynamic stability, and platform mass. They are also the most commercially mature configuration reviewed here.

Boston Dynamics developed BigDog under a DARPA program as an early benchmark for dynamic quadrupedal locomotion. High-power hydraulic actuation and distributed force sensing allowed the robot to carry loads over rocks, snow, and slopes (Figure 3b). Its noise and limited autonomous perception restricted the platform largely to early military transport demonstrations [44]. Spot uses a lighter electric design and combines lidar and inertial sensing with the NeBula autonomy architecture for large-scale GPS-denied mapping in tunnels and disaster sites; Figure 3c shows Spot equipped for autonomous exploration [45]. The system won the urban circuit of the DARPA Subterranean Challenge. A proprioception-only terrain classifier used joint angles, foot penetration, and limb-force signals. After minimum-redundancy maximum-relevance feature selection and principal component analysis, it distinguished concrete, grass, and gravel with 97% offline accuracy [46]. This sensing approach can support operation in darkness and smoke, where vision may fail [45].

The MIT Cheetah series targets highly dynamic running. Seok et al. formulated design principles for energy-efficient legged locomotion and implemented them on the MIT Cheetah [30]; their comparison of cost of transport across terrestrial organisms and mobile robots is reproduced in Figure 3d. Cheetah 3, shown in Figure 3e, combines high-torque-density electric actuation with high-bandwidth proprioceptive control to climb stairs without vision or external terrain sensors [47]. The robot still depends on internal state feedback to close the control loop and should not be described as sensorless. Hardware-control co-design on Mini Cheetah [48], together with robust compliance optimization, reduced the cost of transport in simulation and experiments [49]. Guided reinforcement learning has enabled omnidirectional three-dimensional jumping [50]. A cheetah-inspired quadruped with a three-degree-of-freedom active spine provides a separate test bed for high-speed compliance [51].

ETH Zurich’s ANYmal platform supports dynamic quadrupedal locomotion [52]. Figure 3f shows the first-generation ANYmal platform [52]. In 2025, Rytz et al. proposed a platform-adaptive locomotion method for quadrupeds with different morphologies and dynamics. They trained a single dynamics-conditioned policy by deep reinforcement learning on procedurally generated robots. Tests on ANYmal C (Figure 3g) showed better velocity tracking than conventional approaches and robust zero-shot transfer across multiple previously unseen simulated quadrupeds [53].

Unitree’s Go2 uses a perception-action architecture in a commercial quadruped. Its two-level controller couples learned locomotor behaviors with task planning. With a single depth camera, Go2 traversed randomized obstacle courses, sprinted over hurdles at up to 3.2 m/s, and achieved a 78% completion rate [54]; Figure 3h illustrates the obstacle-course experiment. EPFL’s PAWS platform in Figure 3i takes a different approach: synergistic compliance reproduces several natural quadrupedal gaits while reducing actuator and control complexity [55,56].

The quadrupeds reviewed here range from research platforms to systems demonstrated in field exploration. Their maturity must be judged for the specific configuration and task tested [43,45,47,54]. Validation for another mission, sustained endurance, or a more demanding environment requires separate evidence.

### 3.3. Jumping Robots: High-Power Obstacle Negotiation

**Figure 4:**
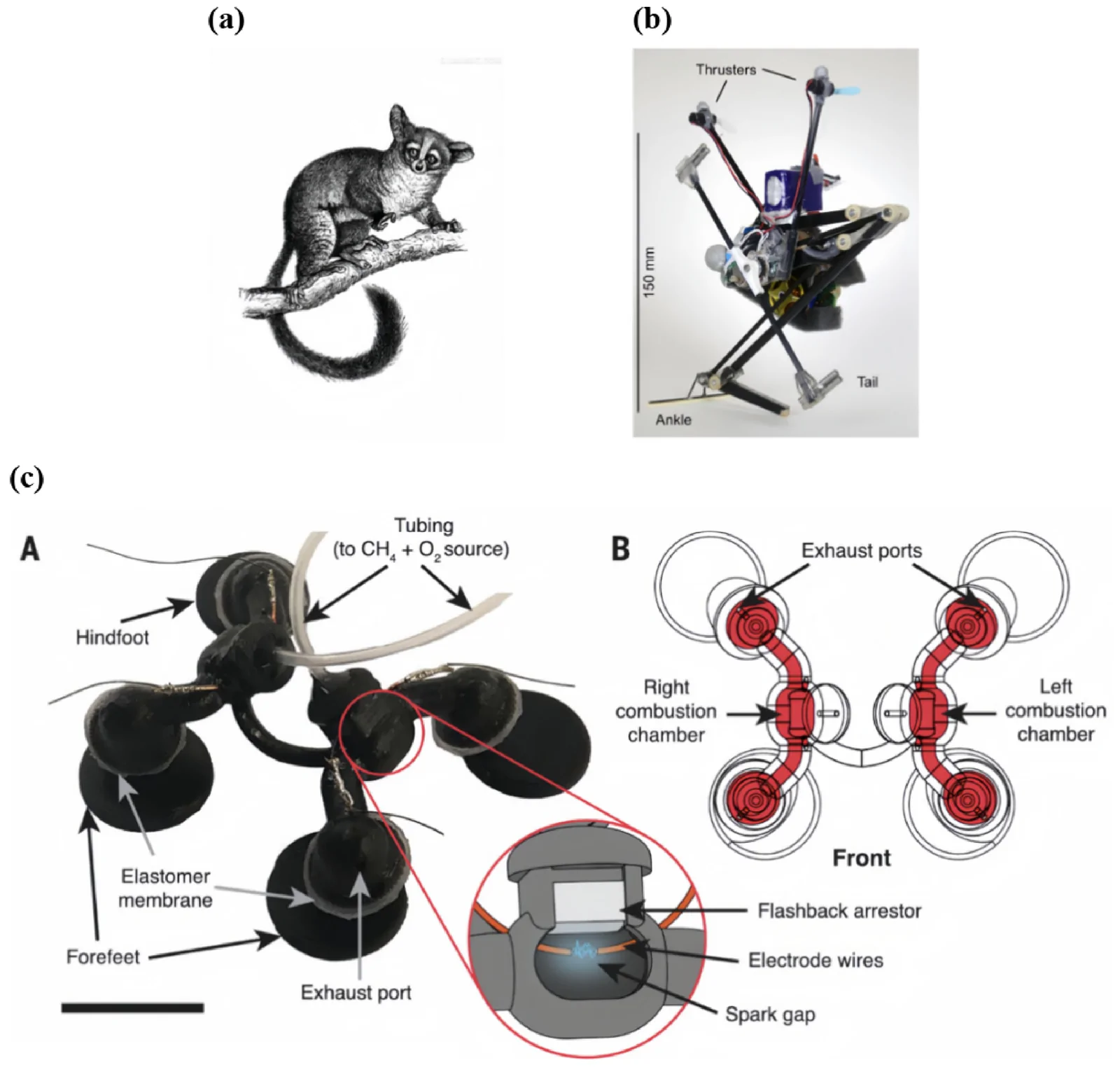
Representative jumping robots. (**a**) Senegal bushbaby, the biological model for branch-to-branch leaping; (**b**) the baseline three-dimensional Salto-1P monopedal robot [57]; and (**c**) an insect-scale quadrupedal microrobot driven by combustion [16].

Jumping robots take inspiration from bushbabies, frogs, and locusts. The Senegal bushbaby in Figure 4a illustrates the biological model for branch-to-branch leaping. Their main design challenge is to store energy efficiently and release it within a short takeoff interval.

The 100 g Salto robot, developed at the University of California, Berkeley and inspired by the Senegal bushbaby, used series-elastic power modulation for agile jumping [57]. Salto-1P later added a reaction wheel for aerial attitude control and performed repeated three-dimensional jumps with peak accelerations of up to 14g; the system was presented at IROS 2017 [57]. Figure 4b shows the Salto-1P platform used for three-dimensional jumping.

At insect scale, soft combustion actuators can provide high instantaneous power. Aubin et al. integrated a compliant combustion actuator into an insect-scale jumping robot (Figure 4c) and achieved rapid takeoff with chemical energy in a compact soft mechanism [16]. The cited study did not demonstrate autonomous landing recovery or closed-loop onboard sensing, so these capabilities remain separate engineering requirements.

Repeated field operation requires three coupled control loops: stored-energy estimation before release, attitude regulation in flight, and contact or force detection at landing. Salto-1P uses aerial attitude actuation and external position feedback, but current jumping prototypes remain limited by intermittent operation, landing robustness, and the small mass budget available for onboard sensing and energy storage [57].

### 3.4. Gliding Robots: Aerial-Transition Locomotion

In robotic gliding, wing geometry, angle of attack, flight path, attitude, and landing energy must be treated as independent states. The EPFL jump-glider uses a 4 mm DC motor, a four-stage gearbox, and a cam to charge two torsion springs, which release through a four-bar leg. A 20 mAh battery supplies onboard energy, while a tail and rudder provide steering. Starting from a height of 2 m, the 16.5 g platform traveled 4.52 m on average, compared with 2.03 m for the wingless jumper, and reduced impact energy by about 54%. The range increase was 123%. Three-channel infrared commands initiated jumping and steering. An external camera operating at 30 frames/s measured the trajectories and did not provide onboard closed-loop sensing [26].

A second robot, inspired by flying squirrels, uses a flexible membrane for terrestrial walking and aerial gliding, with leg and tail motion regulating the angle of attack. It achieved a mean glide ratio of 1.88 and a mean walking speed of 13.38 cm/s [27]. These platforms require mechanisms, actuators, and sensing variables that differ from those of pure jumping systems. Their cited demonstrations establish task-specific jump-glide or walk-glide performance, rather than qualification of a complete field-deployed system.

### 3.5. Climbing and Peristaltic Robots for Confined and Extreme Environments

#### 3.5.1. Climbing Robots

**Figure 5:**
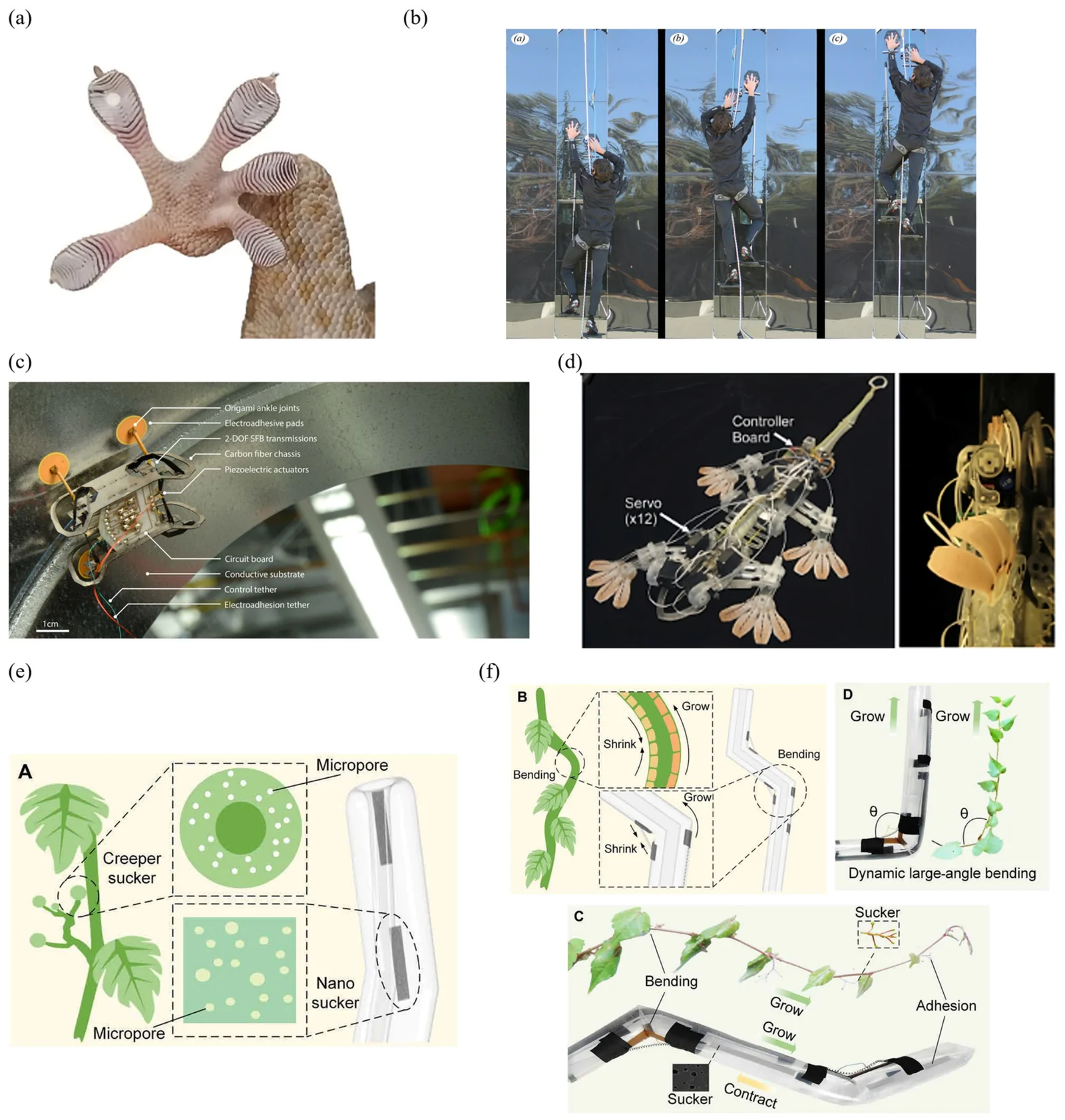
Climbing organisms and robotic platforms. (**a**) Macroscopic view of a tokay gecko foot; (**b**) Stanford’s dry-adhesive climbing system, whose two 140 cm^2^ hand pads can support a 70 kg person on glass [58]; (**c**) the HAMR-E quadrupedal microrobot climbing vertically and upside down [59]; (**d**) a variable-stiffness gecko-inspired robot for space applications [21]; (**e**) the suction structures of Virginia creeper; and (**f**) a growth-mediated soft climbing robot [20].

Gecko-inspired dry adhesion is a well-studied strategy for climbing robots; Figure 5a shows the biological foot structure that motivates this approach. Bioinspired adhesive pads developed at Stanford can support an adult on glass. Hawkes et al. designed a synthetic adhesive system whose load capacity scales with area; two 140 cm^2^ hand pads supported a 70 kg person on a vertical glass surface (Figure 5b) [58]. At a smaller scale, Harvard researchers used electroadhesion to enable the HAMR-E quadrupedal microrobot to climb vertical and inverted surfaces (Figure 5c) [59].

A 2024 study reported a gecko-inspired robot with variable-stiffness adhesive paws (Figure 5d). The robot moved on smooth and rough spacecraft surfaces in microgravity and performed tasks including satellite capture [21]. These tests indicate potential for in-orbit servicing, but they also show that adhesion control must account for substrate condition and load.

Biological models for climbing robots now extend beyond the gecko. The attachment structures of Virginia creeper are shown in Figure 5e. In 2025, Jun Zou’s group at Zhejiang University reported a Virginia-creeper-inspired, growth-mediated climbing mechanism in Science Advances (Figure 5f). The robot grows a microstructured biofilm that strengthens attachment, analogous to adhesive-disc formation in Virginia creeper. This process allows it to negotiate complex, discontinuous surfaces [20].

#### 3.5.2. Peristaltic Soft Robots

**Figure 6:**
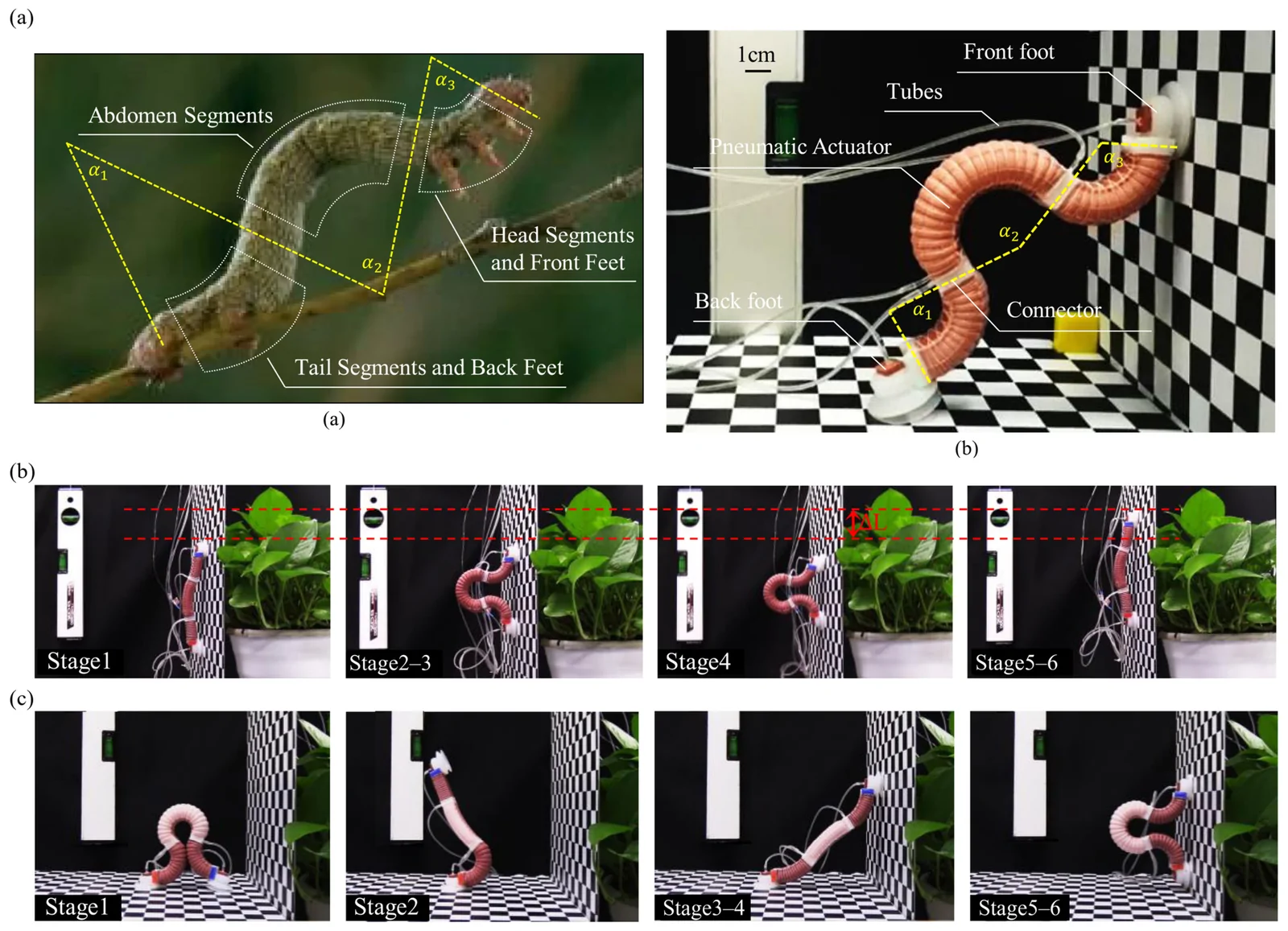
Inchworm-inspired multimodal soft robot. (**a**) Comparison of the inchworm and the complete robot; (**b**) stable vertical-wall climbing; and (**c**) a full gait sequence for autonomous transition from level ground to a wall [23].

Inchworm-inspired soft robots can move on vertical walls and through narrow pipes. Figure 6a compares the biological inchworm with the multimodal platform developed by Zhang et al., which is driven by three pneumatic artificial muscles [23]. It reached 21 mm/s on horizontal surfaces and 15 mm/s on vertical walls. In horizontal locomotion, it carried approximately 500 g, about 15 times its own mass [23]. Figure 6b illustrates vertical-wall climbing. The robot also crawled, climbed, and transitioned from a horizontal surface to a vertical wall, with the transition sequence shown in Figure 6c.

Recent soft-robot studies also decouple actuation from sensing. A multichamber inchworm produces inching and peristaltic motion under 70 kPa pneumatic pressure [60], but the study reports no closed-loop terrain sensor. By contrast, a stimulus-responsive electrofluidic nervous system combines dielectric-elastomer and electroadhesive actuators and reverses a crawling robot in response to touch and heat [61]. In the latter design, the stimulus response is part of the actuation architecture.

Climbing and peristaltic demonstrations remain specific to the tested surface, supply arrangement, speed, and load. Some studies document commanded voltage or pressure but do not establish whether adhesion margin, substrate friction, or body strain was estimated during operation. Relevant feedback or validation quantities include contact force, pressure, strain, and slip, selected for the attachment mechanism and task [21,23,59,61,62].

### 3.6. Composite Locomotion Modes: Multimodal Integration

Composite robots use adaptive morphogenesis to switch between two or more locomotion modes within one body. They commonly combine the speed of wheels with the obstacle-crossing ability of legs. Two main designs are wheel-leg integration and amphibious fin-leg transformation.

The three-spoked Whegs mechanism is a passive composite design in which one component functions as both a rolling wheel and a climbing leg. Terrain-dependent spoke deformation removes the need for a dedicated transformation actuator and simplifies control [63]. In AmphiHex-I, cables deform a compliant steel plate into a cylindrical load-bearing leg on land or a flat propulsive fin underwater [64]. Autonomous switching requires sensing of the environment and configuration state, as well as monitoring of transition energy, but current demonstrations do not report these quantities consistently.

A powered flying-jumping microrobot provides a related boundary case for terrestrial-aerial transitions. It combines four dielectric-elastomer-driven flapping-wing modules operating at 330–400 Hz with a passive telescopic leg. External motion capture at 400 Hz supplied takeoff and landing state feedback, and both power and control remained offboard. The platform regulated jump height from 1.5 to 20 cm at 2–8.4 Hz. Hopping used 64% less power than flight and increased payload capacity tenfold [65]. This example illustrates the dependence of transition performance on the tested support arrangements. Its powered-flight capability is not classified as gliding or used to infer the readiness of terrestrial gliding systems.

### 3.7. Actuation and Sensing Requirements Across Locomotion Modes

Sensor requirements follow the state variables listed in Table 2. Table 3 presents a narrative comparison of selected platforms, rather than an exhaustive systematic review. We included a platform when a primary source described its core mechanism and reported actuation, sensing or control, or quantitative performance. The examples cover the six locomotion modes, with the powered flying-jumping system identified separately as a boundary comparator. The table distinguishes measurements reported in the cited experiments from states that may be needed for feedback or evaluation. ‘Not reported’ means that the cited source did not provide enough information to identify the sensor, feedback signal, or performance measure. It does not establish that a sensor was absent, that sensing was unnecessary, or that endurance was zero. We report source-defined dimensionless measures where available and retain dimensional values when the inputs needed for consistent normalization cannot be established; these heterogeneous results are not combined into one ranking.

The normalized quantities in Table 2 can be used where the source supplies the required measurements or reports the corresponding ratio. Cheetah 3 has a reported minimum CoT of 0.45 during trotting [47], whereas the flying-squirrel robot has a reported mean glide ratio G of 1.88 [27]. These examples quantify transport cost and glide geometry, respectively; neither number ranks one mode against the other. They are source-reported measures rather than values reconstructed from assumed dimensions. The inchworm robot’s horizontal payload of about 15 times its own mass is a further task-specific ratio [23], but it does not measure attachment margin or imply the same payload capacity during climbing.

Other conversions require inputs that cannot be established from the performance records summarized here. Speed in body lengths per second retains units of inverse seconds; conversion to normalized speed also requires locomotor frequency and a compatible reference length. The Froude number and normalized jump height require the stated gravity-referenced leg-length convention; body length and active leg length cannot be substituted without reconciliation. Attachment margin requires usable attachment capacity under the tested surface and contact conditions, not only a successful climb. Wave-body speed mismatch requires wave frequency and wavelength, and normalized transition energy requires supplied energy, moving mass, and fixed reference length. We leave these descriptors uncomputed when these inputs are unavailable rather than infer them from photographs, drive frequencies, or nominal dimensions. The EPFL jump-glider’s total range from an elevated start is also retained in its original form because the reported summary does not isolate the unpowered descent needed for a glide ratio [26].

Table 3 separates actuator commands from reported feedback signals. Some experiments document open-loop timing or externally supplied feedback, whereas others do not describe the sensing architecture in sufficient detail. These cases are not equivalent. The reviewed quadruped studies provide detailed evidence of integrated state feedback; incomplete reporting elsewhere limits conclusions about sensing capability and does not, by itself, justify a lower readiness assessment.

Across walking and running, contact timing and body motion are central feedback states. Spot combines joint-related and inertial information; lidar or depth sensing supports perception-dependent tasks on the selected quadrupeds [45,46,54]. RHex’s cited intrinsic-mobility tests instead use an open-loop clock-driven gait [34]. A terrain label is not a continuous estimate of friction or stiffness [46]. Jumping adds stored-energy, aerial-attitude, and landing-contact states; Salto-1P uses external position feedback, so those trials do not establish a wholly onboard estimator for repeated field jumping [57]. For gliding, the EPFL camera measures trajectories externally, while infrared commands initiate jumping and steering; these observations do not establish onboard estimation of air-relative velocity or angle of attack [26].

Climbing requires attachment-state or load information: HAMR-E’s commanded adhesion voltage and open-loop jet-engine test do not establish online attachment-margin estimation [59]. Peristaltic locomotion requires body-deformation and anchor-phase information, but the inchworm study summarized here reports synchronized pneumatic and vacuum commands without identifying onboard state feedback [23]. Adaptive morphogenesis adds shape, stiffness, and transition-completion states; these feedback details are not consistently specified for the selected transformations [25,64]. This mapping separates demonstrated signals from proposed estimation needs. Unreported states are not assumed to have been unmeasured, and the required subset depends on the task and controller.

Reporting completeness and publication bias are separate limitations. A completeness score requires a predefined set of applicable items and separate treatment of ‘not applicable’ and ‘not reported’; otherwise, differences in study objectives can dominate the score. We retain item-level reporting distinctions without assigning an aggregate score. The selected studies do not provide a common outcome estimate with comparable precision for a conventional funnel-plot analysis. Selection and selective reporting may nevertheless favor successful demonstrations, which limits generalization beyond the documented tests.

## 4. Discussion and Outlook

### 4.1. Technology Readiness Across Locomotion Modes

The reviewed robots differ in the extent and setting of their validation. Following the criteria introduced in Section 3.1, Table 4 summarizes laboratory demonstrations, tests in relevant environments, and selected field applications. We interpret each demonstration using the identified platform and task, the reported test environment, and its power and feedback arrangements where specified. A field trial is distinguished from qualification of a complete system, and missing qualification evidence remains unknown. These descriptions are not certified TRL ratings and do not imply that every platform in a locomotion class has the same maturity.

Quadrupedal robots. The cited platforms span research demonstrations and selected field applications [30,43,44,45,46,47,48,50,51,52,53,54,68]. Go2 couples learned gaits with visual planning [54]; Cheetah 3 and Mini Cheetah combine proprioceptive control with compliance-aware design [30,47,48,49]; and the platform-adaptive ANYmal policy transfers one model across different quadruped morphologies [53]. These results document integrated actuation, sensing, and control for the tested tasks. They do not establish equivalent readiness for every platform, controller, or proposed mission.

Field limitations should be attributed to specific platforms and test conditions. For moving supports, Iqbal et al. demonstrated stable Laikago walking on a pitching treadmill, but observed rebounding and foot slip at contact changes because the planned contact forces temporarily became infeasible [66]. Their analytical solution assumes vertical sinusoidal support motion; the experiment was not an at-sea ship-deck trial. It therefore identifies a control and contact-feasibility challenge relevant to shipboard operation, not a general inability of quadrupeds to operate on ships. In woodland tests with ANYmal C, Frey et al. found that geometric traversability estimates incorrectly classified passable high grass as obstacles. Their visual method reached all eight tested navigation goals, although its single-camera field of view, coarse segmentation, and projection onto imperfect terrain maps limited perception [67]. These findings concern the tested controllers and perception pipelines and do not establish reduced mechanical stability for all quadrupeds in dense forests.

Jumping and gliding robots. Salto-1P demonstrates high-acceleration jumping, although its repeated-hopping experiments still use external position feedback [57]. The EPFL jump-glider and flying-squirrel platform demonstrate that gliding can extend range and reduce landing impact while coupling aerial and terrestrial locomotion [26,27]. Their controlled demonstrations establish these functions under the reported protocols. Direct comparison remains difficult because performance depends on launch height, aerodynamic state, and the measurement and control arrangements. Jump height and range should therefore be interpreted under the protocol reported for each platform.

Multi-legged robots. DASH and RoACH demonstrate gram-scale crawling [31,32], whereas RENS H3 uses hierarchical MPC and force control to improve lateral stability on rugged terrain [39]. The examples span laboratory mechanisms and terrain-specific demonstrations. CRABSTER was designed for seabed operation, so its capabilities do not transfer directly to rubble search and rescue [38,69]. Planetary analog studies have examined coordinated legged teams but do not establish the readiness of heavy-duty terrestrial rescue equipment [43].

Climbing and peristaltic robots. Dry adhesives, microspines, electroadhesion, variable-stiffness paws, and growth-mediated attachment support climbing on different surfaces [19,20,21,58,59]. Inchworm-inspired robots can crawl, climb, and transition while carrying a substantial payload relative to body mass [23]. These task-specific demonstrations do not provide a uniform field-qualification record. Dust tolerance, supply arrangements, attachment-state estimation, and sustained payload operation require assessment for the intended environment. Recent pressure-driven and electrofluidic prototypes do not provide a common benchmark for field endurance [60,61].

### 4.2. Endurance and Locomotion-Specific Sensing Limitations

Endurance remains limited because onboard energy, actuation, sensing, payload, and structural mass compete within fixed budgets. The constraint is acute in miniature jumpers and soft crawlers, which require high peak power, pressure supply, or high voltage while leaving little mass for batteries and sensors [12,16,23,59]. Published studies often report speed or a single maneuver but omit duty-cycle endurance under representative sensing payloads. The heterogeneous results in Table 3 therefore cannot support a common endurance ranking.

Published descriptions do not consistently establish which locomotion-specific states are available to the controller. Useful feedback or validation quantities include contact force and slip for legged locomotion, attachment margin for climbing, strain and contact phase for peristalsis, and attitude and trajectory for gliding. These quantities may be measured directly or estimated; the required subset depends on the task and controller. Spot’s proprioceptive classifier distinguishes concrete, grass, and gravel with 97% offline accuracy but does not estimate continuous stiffness or friction [46]. RENS H3 and Cheetah 3 improve proprioceptive force or state regulation [39,47]. The cited results do not establish a common sensing and evaluation protocol across these modes.

Usable comparisons require common measurement boundaries as well as normalized descriptors. Endurance should be reported as operating time to a predefined energy or task-failure limit, together with initial usable energy, duty cycle, and completed distance or cycles. Cost of transport uses total moving mass, including payload, and a declared travel-distance convention. The supplied-energy boundary must identify whether sensing, computation, launch preparation, and external supply losses are included. Different launch heights or initial stored energies must be reported; electrical transport cost alone cannot fairly rank a passive glide against a level-ground traversal. Payload ratio is payload mass divided by the unloaded, fully equipped robot mass, including onboard energy storage and sensors. The achievable payload must also be associated with a stated task and performance requirement. Tethered operating duration is not equivalent to onboard-energy endurance.

Contact robustness should be reported as successful trials divided by total attempts under a declared surface and disturbance protocol, with fall, detachment, or excessive-slip criteria defined before testing. Transition energy is the supplied energy integrated between specified start and completion events; it should be reported in joules and normalized by mgL, together with transition time and success rate. The energy convention must identify whether net input or gross supplied energy is used and how recovered energy is treated. Comparisons must match or stratify the task, commanded speed, substrate and slope, payload, initial energy state, and power and feedback arrangements. Trial counts and variability distinguish repeatable performance from a single demonstration. These are proposed reporting conditions, not a validated universal benchmark. Mode-specific measures remain necessary, and heterogeneous tasks should not be collapsed into a single cross-mode ranking.

### 4.3. Selected Research Directions for Rigid-Soft Coupling, Embodied Intelligence, and Heavy-Duty Multi-Legged Systems

The directions below are a qualitative synthesis of the selected demonstrations, not an exhaustive or numerically ranked research agenda. We link compliance and morphology co-design to the load-bearing, compliance, and transformation examples in Section 3.1, Section 3.2 and Section 3.6 [25,37,49,56,64]. The sensing distinctions in Section 3.7 motivate closer integration of body mechanics with measured contact and state feedback. Terrain-specific tests of load-bearing hexapods motivate further validation under matched payload and terrain conditions [36,37,39]. These links identify questions for testing; they do not establish that a proposed combination will outperform the reported systems.

Rigid-soft coupling offers an alternative to exclusively rigid linkages. Adaptive morphogenesis and transformable flipper legs allow one structure to change both geometry and function [25,64]. Compliance co-design in Mini Cheetah, heavy-duty linkage-based hexapods, BodyGen, and the synergistically actuated PAWS quadruped indicates that structural compliance can reduce control or energetic demands [37,49,56,70]. Evaluations of morphology change should measure shape, stiffness, and transition work in addition to geometry.

Physical intelligence and morphological computation concern how body mechanics contribute to control [71]. Passive compliance can alter contact response before an explicit controller acts, whereas Spot’s proprioceptive classifier extracts terrain-related information from measured robot signals [46]. These are distinct contributions and should not be conflated. One research direction is to test whether compliant contact mechanics, hierarchical force control such as that used on RENS H3, and a platform-adaptive locomotion policy can be combined without losing state observability or stability [39,53]. Any benefit would require comparison with the component approaches under the same tasks.

Go2 uses a two-level behavior-and-planning controller, whereas ANYmal’s reinforcement-learning policy transfers across platforms [53,54]. Learned policies could also be combined with explicit phase structures, such as central pattern generators and the LLS template, to retain interpretable stability variables while adapting to environmental variation [8].

Heavy-duty terrestrial hexapods warrant further study for load-bearing traversal of irregular terrain. The prototypes in Section 3.1 and RENS H3 provide evidence for load-bearing mechanisms and terrain-specific control, respectively [36,37,39]. RENS H3 combines hierarchical MPC with foot-force compensation for lateral walking [39]. Future tests should examine payload, endurance, and recovery under defined terrain disturbances, with comparable tasks for alternative platforms. This is a proposed validation direction rather than evidence that hexapods are generally superior to quadrupeds or tracked vehicles.

### 4.4. Gait Selection and Walking-Running Transitions

Following the co-design outlook in Section 4.3, gait-transition selection is treated separately because it concerns the choice of limb-coordination and body-dynamics patterns without necessarily changing the underlying morphology. Walking–running transitions occur within the walking/running category and involve different mechanisms from transitions to gliding, interface-mediated climbing, or adaptive morphogenesis. They nevertheless share a task-dependent selection problem: energetic cost, contact feasibility, available feedback, and the cost and stability of the transition must be considered when choosing a locomotor behavior.

An optimal gait is defined relative to a robot, an operating condition, and a performance objective. For steady travel at a prescribed speed, gait-specific cost-of-transport curves can identify energetically favorable candidates, provided that terrain, payload, and energy-accounting boundaries are comparable. Energy efficiency alone, however, does not establish suitability for a task. Shafiee et al. found that a walk-trot transition on flat terrain improved both energy efficiency and viability, whereas transitions from trotting to pronking during gap crossing primarily improved the ability to avoid falls [72]. Thus, the gait with the lowest transport cost need not be the most appropriate on discontinuous terrain. Gait selection should consider energy expenditure together with contact feasibility, disturbance recovery, actuator limits, and task completion. An optimal gait demonstrated under one set of conditions should not be presented as universally optimal.

Increasing speed changes the mechanical demands on the limbs and can favor transitions from pendular walking toward spring-like running mechanics [6,7]. In quadrupeds, these changes interact with footfall coordination, but labels such as trot or gallop should not replace an assessment of center-of-mass dynamics and contact timing. Owaki and Ishiguro demonstrated spontaneous walk-trot-gallop transitions in a physical quadruped by changing a speed-related oscillator parameter while using local load feedback [73]. Their results relate the transitions to energetic cost and stability within the tested platform. The Froude number provides a scale-normalized speed measure when gravity dominates and a comparable characteristic leg length is used [28]; it does not establish a universal switching threshold for different robots. A practical selection rule is to compare feasible walking and running gaits at the target speed under matched terrain and payload conditions, favoring lower transport cost only where stable contacts and actuator limits can be maintained. Contact, inertial, and joint-state feedback should then be used to assess whether the transition itself preserves stable locomotion. The heterogeneous studies reviewed here do not support a single numerical switching threshold.

## 5. Conclusions

For RQ1, within the selected scope, the review identifies six recurring biological principles: redundant support and phase coordination for walking and running; stored-energy release with aerial and landing regulation for jumping; lift-drag and attitude regulation for ground-coupled gliding; interface-specific adhesion for climbing; propagating strain waves combined with alternating anchoring for peristalsis; and geometry or stiffness reconfiguration for adaptive morphogenesis. Dimensionless phase, Froude number, force-to-weight ratio, strain and wave-body speed mismatch, glide ratio, and normalized transition measures describe these principles without fixing a particular robot geometry, provided that reference scales and test conditions are stated [6,7,8,9,14,17,18,19,21,22,23,24,25,26,27,28,29].

For RQ2, the cited validation evidence ranges from task-specific laboratory demonstrations to selected quadrupedal field applications. These records do not support one numerical TRL rating for an entire locomotion class. Many reports also lack enough information to establish the feedback variables used or to compare endurance under representative conditions [23,26,27,39,46,47,54,56,57,59]. This reporting limitation does not demonstrate that a capability is absent. Progress requires validation of the complete morphology-environment-control loop under comparable task protocols. The maturity comparison applies to the selected configurations and reported tests; it does not rank bipedal, humanoid, or sustained-flight systems against the included robots.

## Figures and Tables

**Table 1:** Scope of selected recent reviews and the present synthesis.

Comparison Aspect	Emphasis in Selected Reviews	Treatment in This Review
Locomotion coverage	Jumping [1], soft crawling [2], legged locomotion control [3], and multimodal soft locomotion [5].	Six substrate-dependent modes across representative rigid, soft, and hybrid platforms.
Sensing	Proprioceptive/tactile mechanisms and soft-sensor integration [4]; sensing and control limitations in jumping [1].	Mode-specific feedback states and reported sensor arrangements, distinguishing onboard and external systems where stated.
Actuation	Crawling classified by actuation mechanism [2]; jumping energy storage [1]; soft actuation and sensor compatibility [4].	Actuation choices related to support, impulsive propulsion, attachment, deformation, and mode transitions.
Performance comparison	Jumping performance, normalized acceleration and limb proportions [1]; actuator and locomotion performance [2].	Mode-specific descriptors with reference scales, source-reported normalized examples, and explicit missing-data limits.
Validation and maturity	Control advances toward indoor/outdoor applications [3]; practical challenges for jumping [1] and multimodal soft robots [5].	Evidence interpreted by task, environment, and power/feedback arrangement; no class-wide numerical TRL ranking.

Note: Entries describe the emphasis of each cited review, not the absence of other content. The present synthesis connects these perspectives; it does not claim priority for reviewing sensing, actuation, normalization, or multimodal locomotion.

**Table 2:** Core kinematics and dimensionless descriptors for six locomotion modes and their physical meanings.

Mode	Minimal Kinematic Representation	Dimensionless Descriptors	Physical Meaning	Configuration-Independent Interpretation
Walking/running	Body/foot path: ${r_{i}}^{*}\left(\tau \right)=\frac{r_{i}\left(t\right)}{L}$ Limb phase: $\varphi_{i}\left(t\right)=2\pi frac\left(\frac{t-t_{0,i}}{T}\right)$ Contact duty factor: $\beta_{i}=\frac{T_{contact,i}}{T}$	$\frac{l_{stride}}{L}$ $U^{*}=\frac{\bar{U}}{fL}$ $Fr=\frac{{\bar{U}}^{2}}{gL}$ $CoT=\frac{E}{mgd}$ ${\Delta \varphi }_{ij}\equiv \varphi_{i}-\varphi_{j}$ $\left.(\mathrm{mod}2\pi \right)$ $-\pi \leq {\Delta \varphi }_{ij}<\pi$	Stride ratio: Stride length relative to L. Normalized speed: Mean advance per cycle relative to L (periodic motion only). Fr: Inertial-to-gravitational scale ratio. CoT: Energy per unit weight and travel distance. Phase difference: Signed interlimb phase difference; zero means in-phase coordination.	Describes gait coordination independently of leg number and linkage geometry. Contact, joint, inertial, and force sensing estimate phase and body state.
Jumping	Ballistic COM motion; negligible thrust/drag. Takeoff defines the time origin; z is upward. $\tau_{g}=\frac{t}{T_{g}}$ $x^{*}={x_{0}}^{*}+{u_{x,0}}^{*}\tau_{g}$ $z^{*}={z_{0}}^{*}+{u_{z,0}}^{*}\tau_{g}-\frac{{\tau_{g}}^{2}}{2}$	${u_{0}}^{*}=\frac{u_{0}}{\sqrt{gL}}$ $H^{*}=\frac{H}{L}$; $R^{*}=\frac{R}{L}$ ${t_{f}}^{*}=\frac{t_{f}}{T_{g}}$ ${E_{s}}^{*}=\frac{E_{s}}{mgL}$ ${E_{imp}}^{*}=\frac{E_{imp}}{mgL}$	Normalized launch speed: Launch speed relative to the gravity-based speed scale. Normalized height and range: Jump height and horizontal range relative to L. Normalized flight time: Flight duration relative to the gravitational time scale. Stored-energy ratio: Stored elastic energy relative to mgL. Impact-energy ratio: Landing impact energy relative to mgL.	Permits cross-scale comparison of takeoff, elastic storage, range, and landing. Attitude and contact/force sensing provide feedback during flight and landing.
Climbing	Translation: $p^{*}=\frac{p}{L}$ Orientation: $\theta \left(t\right)$ Contact state: $s_{i}\left(t\right)\in \{0,\;1\}$ Attachment capacity: $F_{cap,tot}=\sum_{i}s_{i}F_{cap,i}$	$A^{*}=\frac{\sum_{i}s_{i}F_{cap,i}}{mg}$ ${M_{A}}^{*}=A^{*}-1$ $U^{*}=\frac{\bar{U}}{fL}$ $\beta_{i}=\frac{T_{contact,i}}{T}$ $\mu_{p}=\frac{m_{payload}}{m_{robot}}$	Capacity-to-weight ratio: Available attachment capacity relative to weight. Attachment margin: Nominal excess capacity above weight; zero at static force balance. This is not a complete stability criterion. Normalized speed: Climbing advance per cycle relative to L (periodic motion only). Duty factor: Fraction of the cycle attached (periodic motion only). Payload ratio: Payload-to-robot mass ratio.	Treats normalized translation separately from orientation. $A^{*}$ is the capacity-to-weight ratio, and ${M_{A}}^{*}$ is the nominal attachment margin. Force/contact, pressure, or adhesion-current sensing detects attachment.
Peristaltic crawling	$\varepsilon \left(\xi ,t\right)=\varepsilon_{0}sin\left(\Psi \left(\xi ,t\right)\right)$ $\Psi \left(\xi ,t\right)=2\pi \left(\frac{\xi }{\lambda }-ft\right)+\varphi_{0}$ Anchor state: $a_{i}\left(t\right)\in \{0,\;1\}$	$\varepsilon_{0}$ $\lambda^{*}=\frac{\lambda }{L}$ $U^{*}=\frac{\bar{U}}{fL}$ $\sigma_{wb}=1-\frac{\left|\bar{U}\right|}{f\lambda }$	Strain amplitude: Peak axial strain. Normalized wavelength: Strain-wave wavelength relative to L. Normalized speed: Body advance per cycle relative to L. Wave-body mismatch: Wave-body speed mismatch normalized by wave speed; not a direct contact-slip measurement.	Describes strain-wave coordination and alternating anchoring. The wave-body speed mismatch is a kinematic proxy, not a direct measurement of contact slip. Strain, contact, and pressure sensing synchronize the wave.
Adaptive morphogenesis	Translation: ${p_{c}}^{*}\left(\eta \right)=\frac{p_{c}\left(\eta \right)}{L}$ Orientation: $\theta_{c}\left(\eta \right)$ Transition progress: $\eta =\frac{t-t_{0}}{T_{\mathrm{tr}}}0\leq \eta \leq 1$	${T_{\mathrm{tr}}}^{*}=\frac{T_{\mathrm{tr}}}{T_{g}}$ $R_{K}=\frac{K_{max}}{K_{min}}$ $R_{A}=\frac{A_{max}}{A_{min}}$ ${E_{tr}}^{*}=\frac{E_{tr}}{mgL}$	Normalized transition time: Duration relative to the gravitational time scale, not a cycle period. Stiffness ratio: Stiffness tuning ratio. Area ratio: Area change ratio. Transition-energy ratio: Transition energy relative to mgL.	Uses a fixed initial length and a measured transition interval. No cycle frequency is assigned to a one-off transition. Shape, load, and environment sensing triggers and verifies switching.
Gliding	Planar still-air motion; z upward; θ is the lifting-surface chord angle. $x^{*}=\frac{x}{L}$; $z^{*}=\frac{z}{L}$ $\gamma =atan2\left(\dot{z},\dot{x}\right)$ α=θ−γ	$C_{L}$; $C_{D}$ $G=\frac{\Delta x}{\left|\Delta z\right|}$ Near-steady glide: $G\approx \frac{C_{L}}{C_{D}}$ ${u_{0}}^{*}=\frac{u_{0}}{\sqrt{gL}}$ ${E_{imp}}^{*}=\frac{E_{imp}}{mgL}$	Lift and drag coefficients: Lift and drag normalized by dynamic pressure times reference area. Glide ratio: Horizontal travel per positive height loss in descent; the lift-to-drag approximation requires steady, straight, unpowered gliding in still air. Normalized launch speed: Launch speed relative to the gravity-based speed scale. Impact-energy ratio: Landing impact energy relative to mgL.	Distinguishes aerodynamic travel from a ballistic jump. $G\approx \frac{C_{L}}{C_{D}}$ applies to steady, straight, unpowered descent in still air. With wind, use air-relative velocity to define flight-path and attack angles.

Note: Cycle frequency, limb phase, and contact duty factor apply only to periodic motion. L is fixed in the stated reference posture; the energy boundary and aerodynamic reference area must be reported. The transition-time reference used here is the gravitational time scale, as defined in Section 2.7. Attachment capacity denotes the usable force component opposing gravity under the stated interface conditions, so the nominal margin is not a complete stability criterion. Strain-wave position and wavelength use the same length coordinate. Stiffness and area ratios require positive denominators and identical measurement conventions.

**Table 3:** Mechanisms, actuation, sensing/control, and reported performance of representative robot platforms.

Class/Platform	Core Mechanism	Actuation	Reported Sensing/Control	Reported Performance and Test Condition	Ref.
Multi-legged: RHex	Six compliant rotary legs; open-loop alternating-tripod gait	One hip motor per leg	Clock-driven gait; no terrain sensing in the reported intrinsic-mobility tests	Up to 1 body length/s; traversed broken terrain and height variations exceeding body clearance	[34]
Multi-legged: RENS H3	Crab-inspired articulated legs for lateral walking	Multi-joint leg actuation	Body-pose MPC plus Cartesian foot-force compensation	Lateral walking was more stable and used less energy than forward walking; no absolute metric reported	[39]
Quadruped: Spot	Electric quadruped with an autonomy payload	Electric joint drives	Lidar and inertial navigation; joint angle, foot penetration, and limb-force features for terrain classification	Concrete/grass/gravel classification: 97% offline accuracy	[45,46]
Quadruped: Cheetah 3	High-bandwidth proprioceptive legs	High-torque-density electric actuation	Proprioceptive feedback; no external terrain sensors in the blind stair experiment	Reported minimum dimensionless CoT = 0.45 during trotting; blind stair climbing demonstrated	[47]
Quadruped: Go2	Two-level gait-and-planning architecture	Electric articulated legs	Single depth camera plus low-level robot-state feedback	Obstacle-course speed up to 3.2 m/s; 78% completion	[54]
Quadruped: PAWS	Synergistic compliant morphology	Synergistic actuation plus passive compliance	Compliant morphology reduces control demand; no sensor-specific metric reported in the cited paper	Multiple natural quadrupedal gaits demonstrated; no common speed or endurance metric reported	[55,56]
Jumping: Salto-1P	Series-elastic power modulation with aerial attitude actuation	Motor-driven elastic leg and aerial attitude actuators	External position feedback in repeated-hopping tests; attitude and leg-state control	0.098 kg; 1.25 m standing leap; >2 m horizontal jumps; 14 g stance acceleration	[57]
Jumping: combustion microrobot	Compliant soft combustion chamber	Chemical combustion actuator	No onboard landing-state feedback reported in the cited work	High peak-power jumping demonstrated; protocol did not establish repeated autonomous landing	[16]
Gliding: EPFL jump-glider	Spring jump followed by fixed-wing glide; tail and rudder steering	DC motor, four-stage gearbox, torsion springs; 20 mAh LiPo	Three-channel infrared command; external camera at 30 frames/s for trajectory measurement	16.5 g; from 2 m: 4.52 m versus 2.03 m without wings; impact energy reduced by about 54%	[26]
Gliding: flying-squirrel robot	Flexible membrane; legs and tail regulate angle of attack	Motor-driven legs with compliant membrane	Leg/tail motion controls attitude and angle of attack	Reported mean glide ratio G = 1.88 in gliding tests; mean walking speed 13.38 cm/s	[27]
Boundary comparator: powered flying-jumping robot	Four flapping-wing modules plus a passive telescopic leg	Dielectric elastomer actuators at 330–400 Hz; offboard power	External motion capture at 400 Hz; offboard control	Jump height 1.5–20 cm at 2–8.4 Hz; hopping used 64% less power and carried 10 times the payload of flight	[65]
Climbing: SpinybotII	Compliant microspines engage hard-wall asperities	Motorized climbing chassis	No sensing or feedback modality reported in the available primary source	Climbed concrete, brick, stucco, and masonry; carried its own weight and clung without power	[19]
Climbing: HAMR-E	Electroadhesive pads and passive-alignment ankles	Leg actuation plus voltage-controlled electroadhesion	Commanded leg motion and adhesion voltage; jet-engine test used open-loop control	1.48 g; at 250 V: 1.2 mm/s vertical, 4.6 mm/s inverted; 215/162-step runs	[59]
Peristaltic: inchworm SCCR	Omega-shaped body plus alternating vacuum anchoring	Three pneumatic artificial muscles; two vacuum suckers	Synchronized body deformation and vacuum-sucker commands; no onboard state sensor reported	21 mm/s horizontal; 15 mm/s vertical; 500 g horizontal payload (about 15 times the robot’s own mass)	[23]
Multimodal: AmphiHex-I	Cable-driven compliant plate that switches between a load-bearing leg and a fin	Cable-driven shape change	Mode-state and environment sensing not specified in the cited study	Transitions demonstrated on land, in shallow water, and across mudflats; switching energy and time not reported	[64]

Note: ‘Not reported’ denotes missing information in the cited source, not confirmed absence of a capability. External measurement, remote command, and onboard closed-loop feedback are distinguished where the source permits. The powered flying-jumping system is a boundary comparator and is excluded from conclusions about gliding maturity.

**Table 4:** Comparative characteristics of six terrestrial bioinspired robot configurations.

Robot class	Primary Biological Model	Main Advantage	Main Limitation	Reported Validation	Principal Applications
Multi-legged	Insects and crustaceans (cockroaches, crabs)	Redundant-contact support in selected terrain-tested platforms; high payload potential	Few heavy-duty terrestrial products; low speed and high energy use	Laboratory and terrain-specific tests	Deep-sea exploration, mining, and search and rescue in extreme terrain
Quadrupedal	Quadrupedal mammals (dogs, cats)	Integrated mobility, perception, and control demonstrated in selected platforms; commercial availability of some systems	Platform-specific contact-control limits on moving supports [66] and vegetation-related traversability-estimation errors [67]; task-dependent endurance	Research and selected field demonstrations	Debris search and rescue, power-plant inspection, security patrol, and planetary exploration
Jumping	Frogs, locusts, and bushbabies	Jumps of several body heights demonstrated in selected platforms	Limited sustained operation; risk of overturning on landing	Controlled-environment demonstrations	Planetary-pit exploration and short-range special reconnaissance
Climbing/peristaltic	Geckos, earthworms, and Virginia creeper	Vertical-surface or confined-space access demonstrated in selected attachment and body-deformation designs	Low speed; sustained outdoor reliability not established by the cited tests	Task-specific laboratory demonstrations	Pipeline and facade inspection; rubble-gap search and rescue
Multimodal composite	Turtles and insects	Wheel-leg traversal or land-water transitions demonstrated in selected designs	Mode transformation adds mass and consumes energy; autonomous switching and transition cost are not consistently reported	Selected land-water transition tests	Amphibious reconnaissance and supply transport over complex terrain
Gliding	Flying squirrels and gliding insects	Increased range and reduced landing impact relative to a wingless jumper in the reported EPFL tests	Performance depends on launch conditions and aerodynamics; field endurance and landing robustness are not established by the cited tests	Controlled launch and glide tests	Pit crossing, short-range rubble reconnaissance, and terrain transitions

Note: Advantages and validation descriptions summarize selected cited platforms and test conditions, not all members of each class. Potential benefits are not established capabilities of every platform. These descriptions are not formal TRL assessments; the powered flying-jumping boundary comparator is excluded from the gliding category.

## Data Availability

The data presented in this study and the source code are available on request from the corresponding author due to privacy reasons.
